# Automated chest X-ray disease screening using large language models and deep convolutional neural networks on the MIMIC-CXR dataset

**DOI:** 10.3389/fdgth.2026.1883337

**Published:** 2026-09-07

**Authors:** Qingyu Zhang, Pardeep Vasudev, Kezhi Li, Holger Kunz

**Affiliations:** 1 Institute of Health Informatics, University College London, London, United Kingdom; 2 Department of Primary Care and Public Health, Imperial College London, London, United Kingdom

**Keywords:** CNN, deep learning, disease screening, LLM, medical imaging, MIMIC-CXR, radiology report analysis

## Abstract

**Introduction:**

Radiologists in resource-limited settings often face high workloads, especially in chest X-ray interpretation. Manual annotation of large-scale imaging datasets remains costly and time-consuming. This study aims to explore the feasibility of using large language models (LLMs), specifically GPT-4o, to generate binary disease presence labels from free-text radiology reports, and to use these labels to train deep learning models for automated chest X-ray classification.

**Methods:**

A two-stage supervised learning pipeline was developed using the publicly available MIMIC-CXR v2.1.0 dataset. First, GPT-4o was prompted with a structured clinical protocol to classify each radiology report as either “diseased” or “no disease.” Second, the generated labels were used to supervise the training of four convolutional neural networks: ResNet-18, DenseNet-121, EfficientNet-B1, and ConvNeXt-Tiny. A patient-level 70/10/20 split was employed to prevent data leakage across sets. Each model was trained across five random seeds (42–46), and 95% confidence intervals were computed using the t-distribution. Label quality was evaluated by comparing 210 generated labels against radiologist annotations from a board-certified radiologist.

**Results:**

GPT-4o achieved an overall accuracy of 92.9% with expert labels on the 210-report validation set. For the “diseased” class, the precision was 97.4% and recall was 90.5%; for “no disease,” precision was 87.1% and recall was 96.4%. Among the CNN models evaluated on the held-out test set, ConvNeXt-Tiny achieved the highest area under the curve (AUC=0.832, 95% CI [0.801, 0.863]) and balanced accuracy (0.739), significantly outperforming EfficientNet-B1 (AUC=0.797; paired t-test, p=0.014). ResNet-18 (AUC=0.822) and DenseNet-121 (AUC=0.808) showed intermediate performance. All models demonstrated AUC values above 0.79, confirming the viability of LLM-derived weak supervision.

**Discussion:**

This study demonstrates that LLMs can be effectively employed to generate supervision labels for medical imaging tasks. The proposed approach offers a scalable and low-cost solution for preliminary disease screening, particularly in healthcare environments with limited expert availability. The multi-seed evaluation with confidence intervals provides a rigorous assessment of model stability. Further work is needed to improve label reliability and expand to multi-label classification.

## 1 Introduction

### 1.1 Clinical motivation and diagnostic challenges

Chest radiographs (CXRs) are among the most frequently used diagnostic imaging modalities in clinical medicine, particularly for identifying cardiovascular and pulmonary conditions such as pneumonia, pleural effusion, and heart failure (1). However, interpreting CXRs requires specialised expertise and consistency—resources that are often stretched thin in high-throughput or resource-constrained healthcare systems. These challenges are not limited to low-income countries; in the UK, radiologist shortages are a well-documented concern (2).

**Figure 1:**
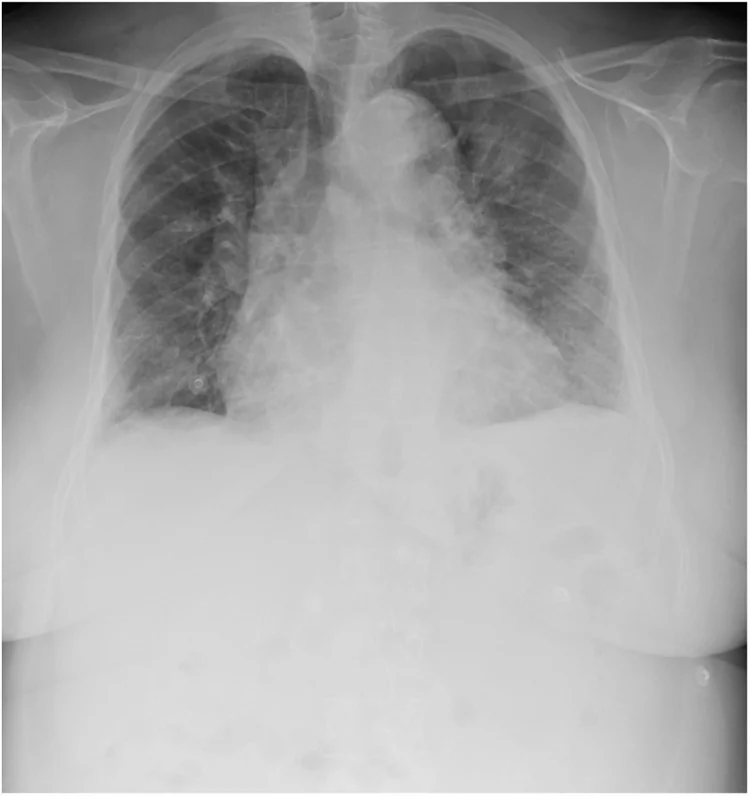
X-ray of diseased chest. Cite mimic cxr V2.1.0.

**Figure 2:**
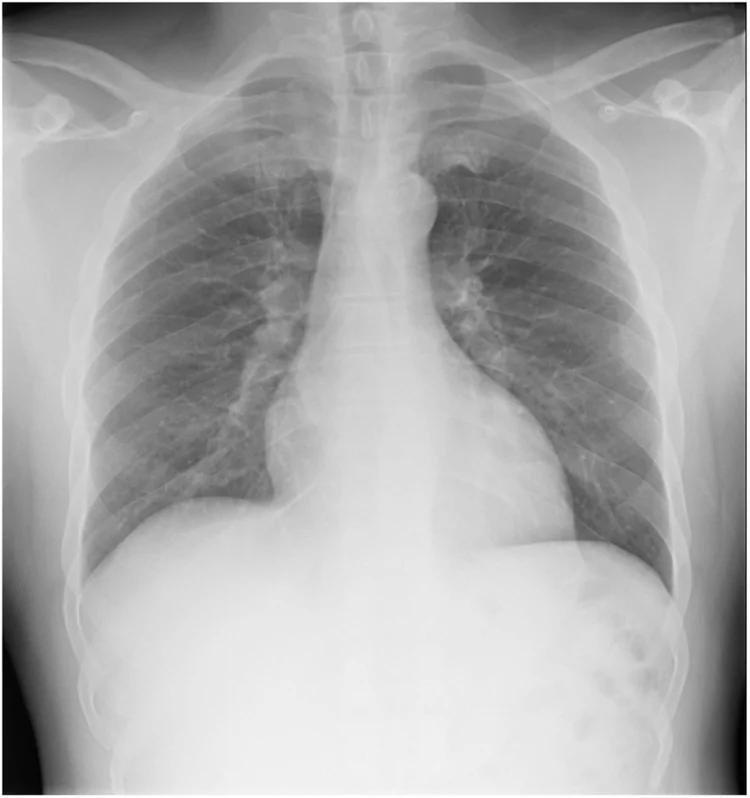
X-ray of healthy chest. Cite mimic cxr V2.1.0.

The interpretation of CXRs is further complicated by the inherent limitations of the imaging modality. As a two-dimensional projection of three-dimensional anatomy, CXRs suffer from the superimposition of anatomical structures, which may obscure key findings. For example, pulmonary nodules may be hidden behind the heart or ribs, and subtle pathologies such as early-stage pneumonia can easily be missed. Moreover, the same image may exhibit multiple overlapping disease patterns, requiring considerable diagnostic acumen. Substantial inter-observer variability among radiologists has also been reported, underscoring the subjectivity involved in interpretation (3). Examples of diseased and healthy chest X-rays are shown in Figures 1 and 2, respectively.

### 1.2 Deep learning for chest X-ray interpretation

Artificial intelligence (AI), and in particular deep learning (DL), has emerged as a promising solution to these diagnostic challenges. Convolutional neural networks (CNNs) have achieved strong performance in various medical imaging tasks, such as breast cancer detection (4, 5), cardiac function analysis (6), and skin lesion classification (7, 8). In the domain of chest radiography, notable work such as CheXNet demonstrated that a 121-layer DenseNet could match radiologist-level performance in pneumonia detection (9).

Transfer learning has played a central role in advancing DL-based CXR interpretation. Most successful models are pre-trained on large-scale natural image datasets such as ImageNet and later fine-tuned on domain-specific CXR data (10, 11). This approach enables the reuse of low-level visual features (e.g., edges, textures) and allows for effective training even when labelled medical data is limited.

However, the development of high-performing models relies heavily on high-quality annotated datasets—something that is often lacking in the clinical domain. Manual annotation is time-consuming and expensive, while rule-based natural language processing (NLP) for label extraction frequently suffers from poor generalisation due to the variability of clinical language.

### 1.3 Large language models in clinical NLP

Recent advances in large language models (LLMs), such as GPT-4, PaLM, and LLaMA, offer a compelling alternative to traditional NLP pipelines. Built on the Transformer architecture (12), these models are capable of understanding and generating human-like text with remarkable contextual accuracy (13–15).

LLMs can be categorised as encoder-based (e.g., BERT), decoder-based (e.g., GPT-3), or encoder-decoder-based (e.g., T5), and are typically trained on massive corpora using self-supervised objectives. As LLMs have scaled in size and training data, their zero-shot and few-shot learning abilities have improved dramatically (16), allowing them to perform new tasks with minimal or no task-specific tuning.

In healthcare, LLMs have been applied to tasks such as clinical summarisation, ICD code prediction, radiology report generation, and patient education through medical chat interfaces (17–19). When combined with prompt engineering, LLMs can extract structured labels from unstructured clinical text without additional training (20).

This study leverages GPT-4o to extract binary labels (diseased/no disease) from free-text CXR reports. Previous approaches to label generation, such as CheXpert (21), focused on diagnostic systems, whereas our approach focuses solely on screening for clinical relevance. While this approach offers scalability, it also introduces potential risks, such as label noise, bias, and lack of transparency. Prior work has emphasised the importance of uncertainty quantification and model calibration when using LLM-generated labels in clinical contexts (22).

### 1.4 CNN architectures for chest X-ray classification

CNNs remain foundational in medical imaging due to their inductive biases and computational efficiency. This study evaluates four widely used CNN architectures:

- **ResNet-18:** Incorporates residual skip connections to mitigate vanishing gradients and enables deep model training (23).
- **DenseNet-121:** Connects each layer to every preceding layer, promoting feature reuse and facilitating gradient flow (24).
- **ConvNeXt-Tiny:** A modern CNN architecture inspired by Vision Transformers, using large kernels, GELU activations, and layer scaling (25).
- **EfficientNet-B1:** Employs compound scaling to optimise model depth, width, and resolution (26).

These models offer a range of architectural choices and inductive biases, making them suitable for comparative analysis under a supervised learning paradigm.

### 1.5 Study objectives and contributions

This study proposes a scalable two-stage pipeline to support automated chest X-ray classification for clinical screening purposes:

1. Data Pre-processing: Use GPT-4o to extract binary disease/no-disease labels from radiology reports in the MIMIC-CXR dataset for label generation;
2. Image Classification: Train CNN models using the LLM-generated labels to classify CXR images.

This approach aims to reduce reliance on manual annotation and rule-based NLP, while exploring the reliability and generalisability of LLM-generated labels in a clinical setting.

## 2 Materials and methods

### 2.1 Dataset and subset selection

#### 2.1.1 Dataset description

This study utilises the MIMIC-CXR v2.1.0 dataset, a large-scale, publicly available chest radiograph dataset released by the MIT Laboratory for Computational Physiology (27). The dataset contains 377,110 de-identified chest radiographs and 227,835 corresponding free-text radiology reports collected from 65,379 patients admitted to the Beth Israel Deaconess Medical Center between 2011 and 2016. Each radiograph is stored in DICOM format, and each report is linked to one or more images via a unique study identifier.

All data are accessible under a credentialed data use agreement, and the dataset has been ethically reviewed and approved. MIMIC-CXR is widely used in research focused on computer vision, NLP, and multimodal learning in medical imaging.

#### 2.1.2 Subset selection

The selected subset includes:

– **623 unique patients**, each stored in a separate folder (e.g., p10/p10000032);
– **2,105 radiographic studies**, each in a subfolder (e.g., s50414267), representing a single imaging encounter;
– **2,105 corresponding radiology reports** in plain-text format.

Each study folder contains one text file (view*.txt) storing the full radiology report, and one or more DICOM images named using view position codes (e.g., PA, AP, LATERAL).

#### 2.1.3 Image modalities and view types

The subset includes multiple view types, with the majority of images being either posterior-anterior (PA) or anterior-posterior (AP) projections. A smaller proportion of images are lateral views. Multi-view studies typically contain both frontal and lateral images. The distribution of view types is consistent with those reported in the full dataset.

**Figure 3:**
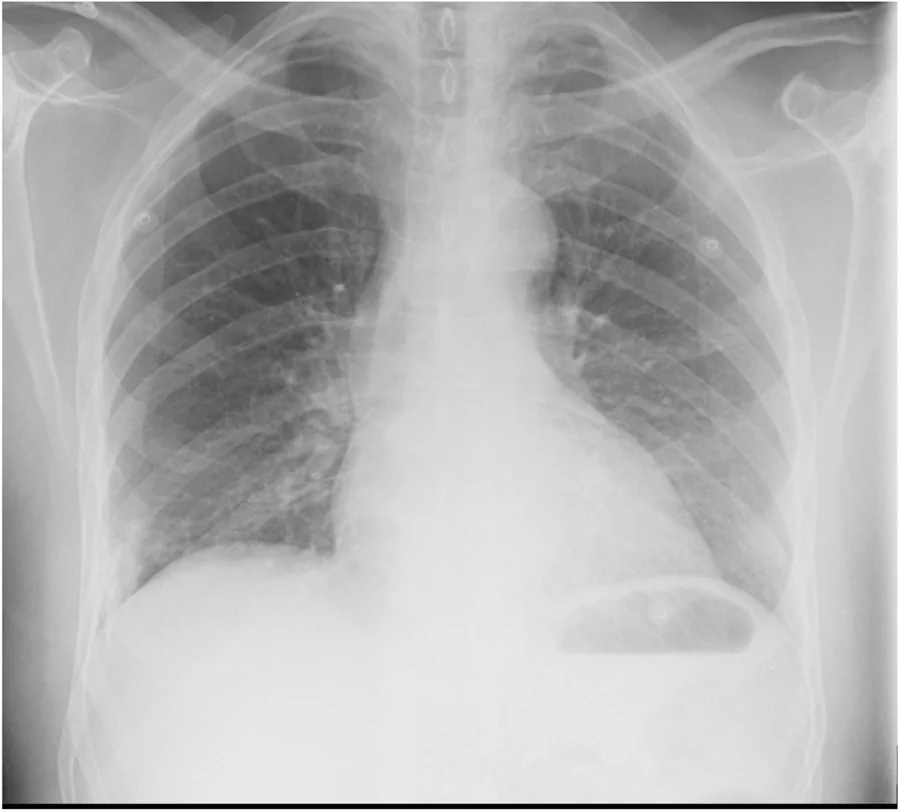
Example chest X-ray image. Cite mimic cxr V2.1.0.

All images remain in their original DICOM format and resolution at this stage. No resizing or normalisation is applied prior to preprocessing. Figure 3 shows an example chest X- ray image from the dataset.

#### 2.1.4 Radiology report structure

Each report consists of unstructured free-text, typically divided into sections such as:

– FINDINGS: Description of observed features in the radiograph;
– IMPRESSION or CONCLUSION: Summary diagnosis or clinical interpretation;
– Occasional subheaders like INDICATION and TECHNIQUE.

The structure is not strictly standardised across all reports, and some reports may omit certain sections. Most reports are relatively concise (typically 50–200 words) and written in domain-specific clinical language. Table 1 presents an example radiology report from the dataset, illustrating the typical section structure.

**Table 1:** Example radiology report.

Section	Content
History	Dyspnoea and history of lung cancer.
Technique	Semi-upright anteroposterior (AP) view of the chest.
Comparison	CT torso and chest radiograph.
Findings	Lung volumes are low, resulting in crowding of the bronchovascular structures. There may be mild pulmonary vascular congestion. The heart size is borderline enlarged. The mediastinal and hilar contours are relatively unremarkable. Innumerable nodules are demonstrated in both lungs, more pronounced in the left upper and lower lung fields, compatible with metastatic disease. No new focal consolidation, pleural effusion, or pneumothorax is seen. Chronic elevation of the right hemidiaphragm is again noted. The patient is status post right lower lobectomy. Rib deformities within the right hemithorax are compatible with prior postsurgical changes.
Impression	Innumerable pulmonary metastases. Possible mild pulmonary vascular congestion. Low lung volumes.

#### 2.1.5 Data integrity and preprocessing decisions

During dataset preparation, a small number of studies were excluded based on the following criteria:

– Missing or corrupted DICOM files;
– Empty or incomplete radiology reports;

After filtering, 2,097 high-quality studies remained, all of which include at least one frontal-view image and a complete report.

This curated subset allows for controlled experimentation while preserving the real-world complexity of CXR interpretation, including multi-view imaging, variable report structure, and diverse pathology prevalence.

### 2.2 Study design

**Figure 4:**
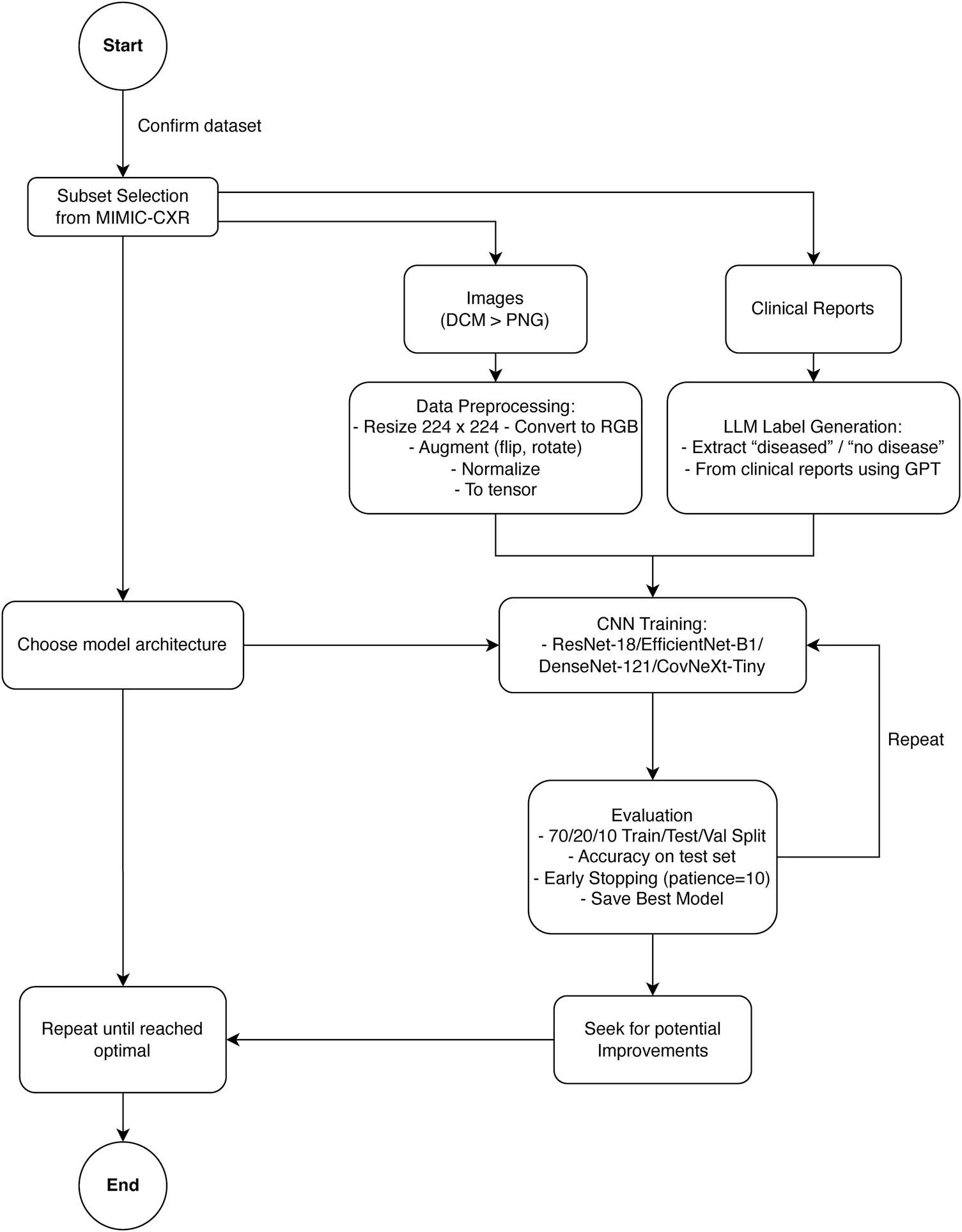
Study design pipeline.

This study follows a two-stage pipeline. In the first stage, binary labels (“diseased” or “no disease”) are automatically extracted from radiology reports using a large language model (LLM). In the second stage, these labels are used to train convolutional neural networks (CNNs) to classify chest radiographs. The overall study design is illustrated in Figure 4.

#### 2.2.1 Technical specification

The deep learning models in this study were developed and trained locally on an ASUS ROG Zephyrus M16 GU604VI (ASUSTeK Computer Inc., Taipei, Taiwan), equipped with a 13th Generation Intel^®^ Core^™^ i9-13900H processor (Intel Corporation, Santa Clara, CA, USA) running at 2.60 GHz, 32 GB of RAM, and an NVIDIA GeForce RTX 4,070 Laptop GPU with 8 GB of GDDR6 memory (NVIDIA Corporation, Santa Clara, CA, USA).

All experiments were conducted within a dedicated Conda environment configured on a 64-bit Windows operating system, with Python 3.8.20. The models were implemented using PyTorch, an open-source machine learning library developed by Facebook’s AI Research lab, which offers a flexible and dynamic computational graph along with support for CUDA-enabled GPU acceleration. Specifically, PyTorch version 2.4.1, compiled with CUDA 12.1 support, was used in conjunction with TorchVision 0.19.1 and TorchAudio 2.4.1, enabling seamless integration of image and audio processing capabilities. Visual Studio Code (Microsoft Corporation, Redmond, WA, USA) was employed as the integrated development environment (IDE), providing an efficient and reproducible workflow for model development, training, and evaluation.

#### 2.2.2 Preprocessing

A consistent data preprocessing pipeline was applied to all CXR images prior to model training, with the aim of standardising inputs, improving generalisability, and preventing overfitting.

- **Image Standardisation:** DICOM images were converted to PNG and resized to 224×224 pixels (240×240 for EfficientNet) using bilinear interpolation. Greyscale images were converted to RGB channels to meet model input requirements.
- **Data Augmentation:** Randomised transformations were applied during training to simulate clinical variability:
  1. Horizontal flipping (50%)—simulates left/right orientation differences.
  2. Rotation ($\pm 10^{\circ }$)—accounts for variations in positioning.
  3. Colour jitter—introduces brightness and contrast noise to simulate hardware variability.
- **Tensor Conversion and Normalisation:** Images were converted using PyTorch’s ToTensor() and normalised using standard ImageNet statistics:
  ∘ Mean=[0.485,0.456,0.406].
  ∘ Std=[0.229,0.224,0.225].
- **Dataset Filtering and Splitting:** Only samples with valid images and binary labels were retained. A patient-level split was performed with approximately 1,468 studies for training (70%), 210 for validation (10%), and 419 for test (20%), ensuring that all reports from the same patient appear exclusively in one subset to prevent data leakage. For each random seed, the split was re-generated while maintaining the patient-level constraint and label stratification.

#### 2.2.3 Stage 1: label generation via large language model (LLM)

To derive weak supervision labels from the MIMIC-CXR radiology reports, the gpt-4o model from OpenAI was employed. GPT-4o is a multimodal transformer-based large language model capable of processing both text and images. Its application in this context allowed for scalable and consistent textual labelling of free-form medical reports, with improved speed and cost-effectiveness compared to earlier GPT-family models.

**Figure 5:**
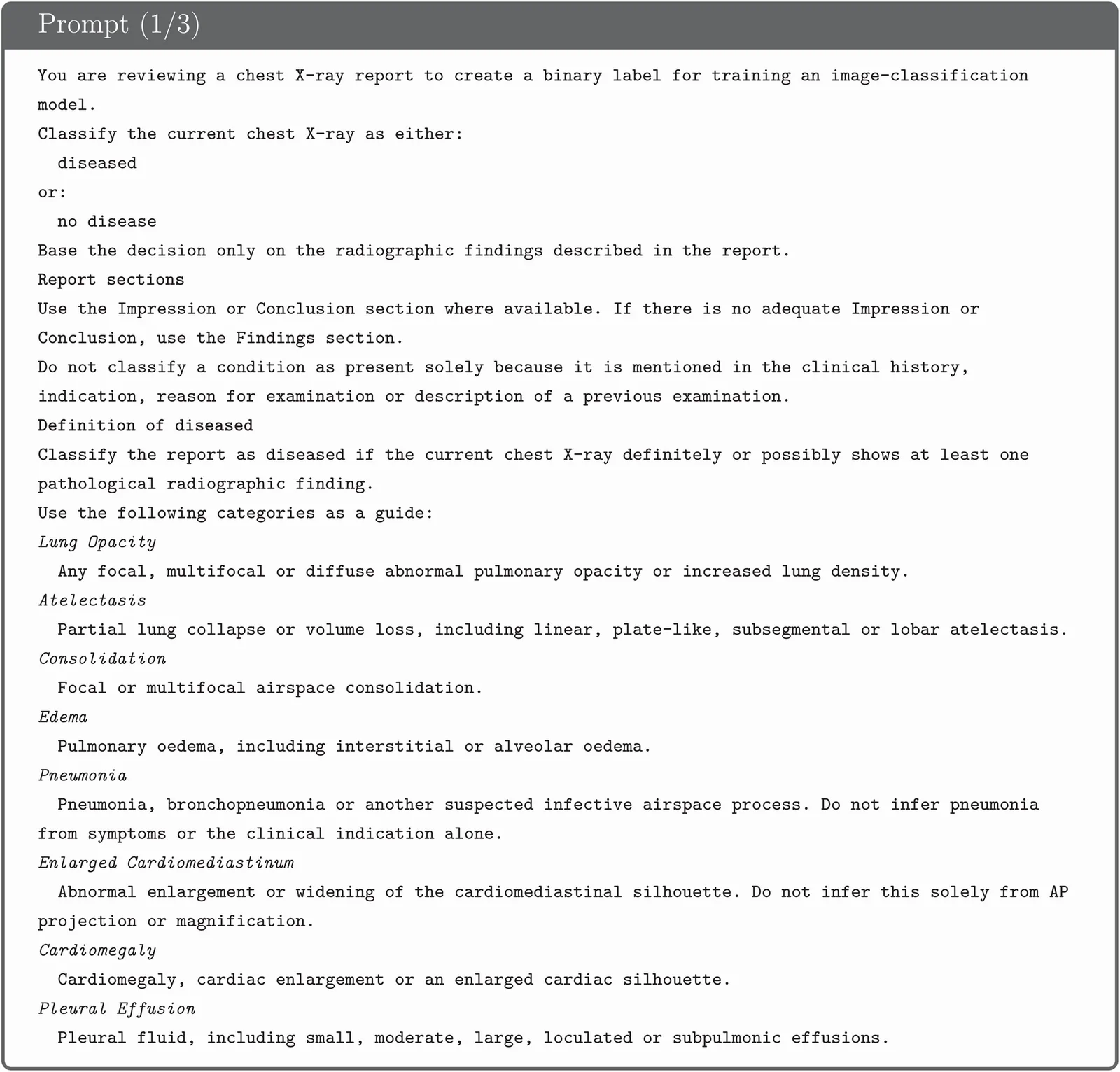
Structured clinical prompt (part 1 of 3): opening instructions, source sections, definition of “diseased,” and the first eight radiographic finding categories.

**Figure 6:**
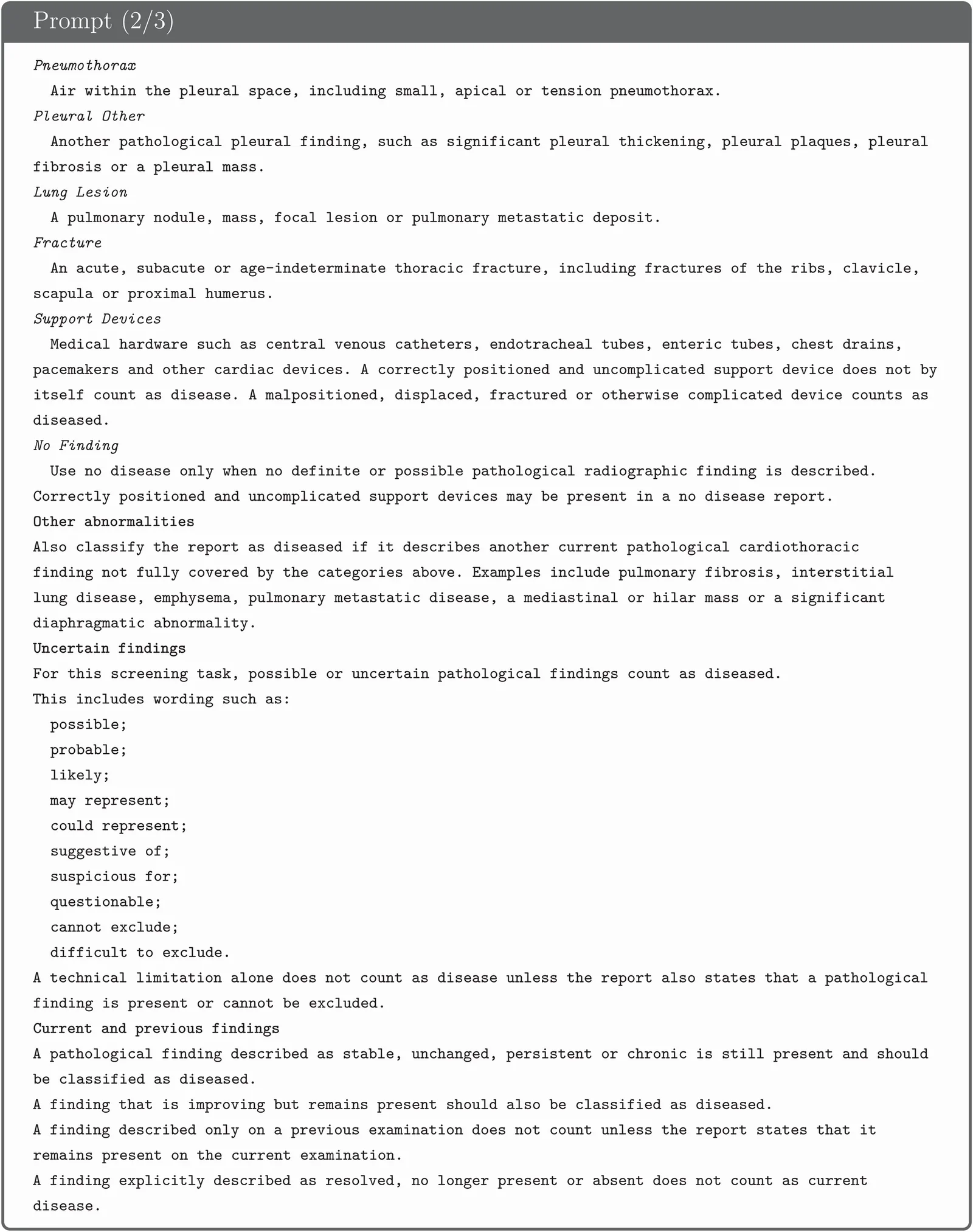
Structured clinical prompt (part 2 of 3): remaining six finding categories, other abnormalities, handling of uncertain findings, and rules for current vs. previous findings.

**Figure 7:**
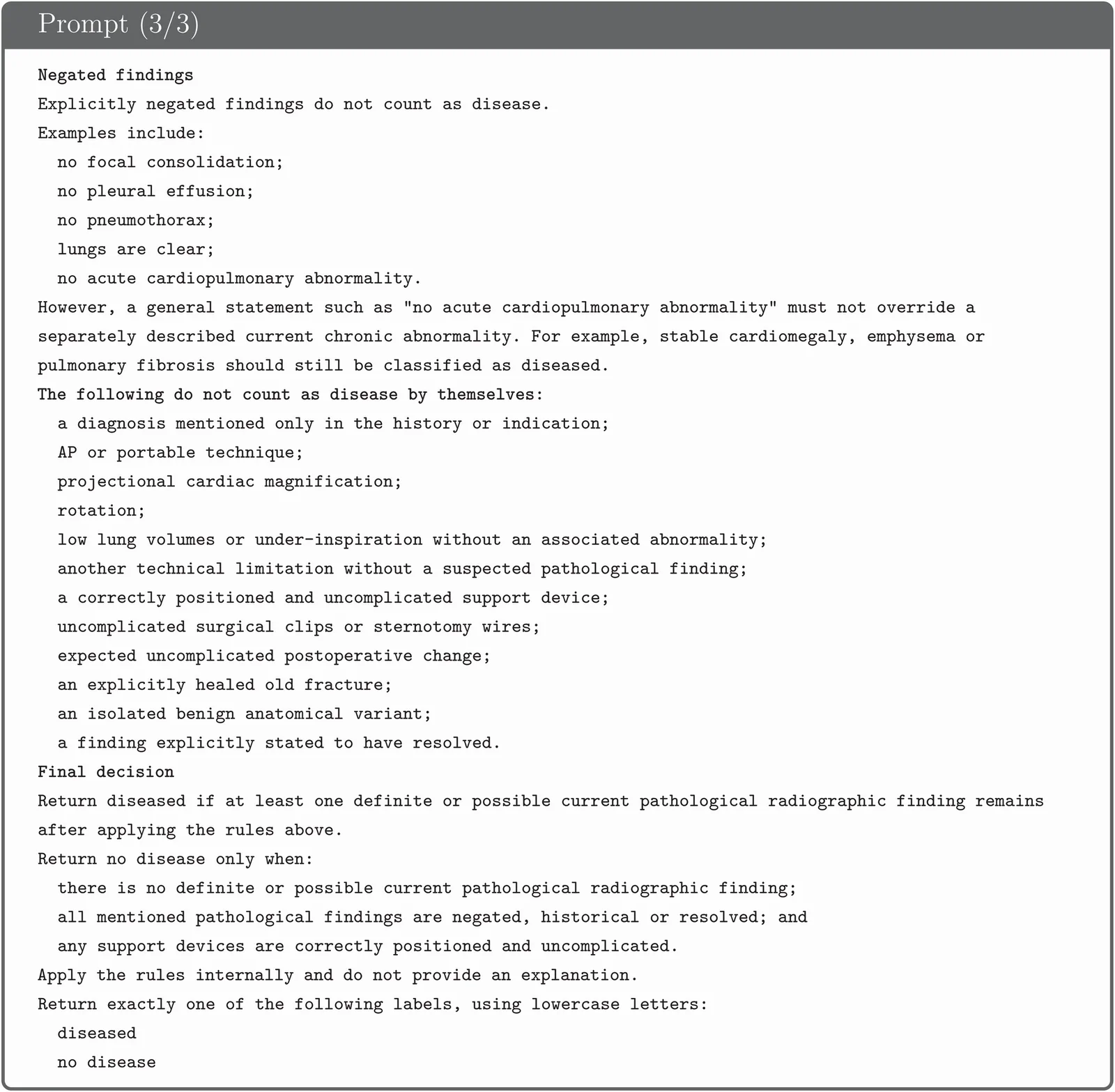
Structured clinical prompt (part 3 of 3): negated findings, conditions that do not count as disease, and the final decision protocol.

Each chest X-ray report was processed individually using a deterministic prompting strategy. A structured clinical protocol, refined in collaboration with a board-certified radiologist, was designed to guide the classification. The protocol defined “diseased” as the presence of at least one definite or possible pathological radiographic finding across 14 anatomical and pathological categories (including lung opacity, atelectasis, consolidation, edema, pneumonia, cardiomegaly, pleural effusion, pneumothorax, lung lesion, fracture, and support devices with complications). It specified that uncertain findings (e.g., “possible,” “likely,” “cannot exclude”) count as diseased, while negated findings, historical abnormalities, and uncomplicated support devices do not. The model was instructed to return exactly one of two labels using lowercase letters: diseased or no disease. The full prompt is shown in Figures 5–7.

API queries were conducted using a temperature setting of zero to ensure deterministic responses, with up to five retry attempts and exponential backoff implemented in case of transient API failures or rate limiting. The model’s output was parsed and normalised to lowercase. Outputs containing the expected substrings “diseased” or “no disease” were retained as valid labels.

The labelled results, comprising patient identifiers, report filenames, and predicted labels, were stored in a structured CSV file (mimic_all_labels.csv). This file subsequently served as the primary annotation source for downstream image-based classification. No dynamic prompt tuning or few-shot examples were employed. Although the current method does not incorporate uncertainty quantification, future extensions may include ensemble prompting, confidence calibration, or filtering based on response consistency to enhance label reliability.

##### 2.2.3.1 LLM Label Evaluation

To assess the reliability of these automatically generated labels, we conducted a validation study against expert annotations. A set of 210 CXR reports was randomly sampled and independently labeled by a board-certified radiologist, serving as the reference standard. The expert-labeled dataset contained 126 diseased and 84 no-disease reports.

We then compared the LLM-generated labels to the expert annotations using standard classification metrics, including precision, recall, F1 score, and overall accuracy. A detailed breakdown of this evaluation is presented in Section: Results and Analysis.

#### 2.2.4 Stage 2: CNN-based image classification

The binary image-level labels generated by the LLM were subsequently used to train convolutional neural networks (CNNs) to classify chest radiographs as either “diseased” or “no disease”. Four CNN architectures were selected based on their performance in prior literature and their diversity in architectural paradigms:

##### 2.2.4.1 Modified ResNet-18 architecture

This study adopt the ResNet-18 architecture as the backbone for chest X-ray image classification, leveraging its proven effectiveness in medical imaging tasks while maintaining a lightweight footprint suitable for large-scale training. The base model is initialized with ImageNet-pretrained weights and subsequently fine-tuned on our dataset. Several key modifications are applied to adapt it to the binary classification task (“diseased” vs. “no disease”). These modifications are summarised in Table 2:

**Table 2:** Summary of ResNet-18 modifications for chest X-ray classification.

Component	Modification
Final FC Layer	Replaced with Dropout(0.5) + Linear(2)
Loss Function	CrossEntropyLoss with class weights
Class Imbalance Handling	Weighted loss via class frequency
Data Augmentation	Resize, Flip, Rotation, ColorJitter, Normalize
Optimizer	AdamW ($\text{lr}=1e^{-4}$, $\text{weight decay}=1e^{-5}$)
Early Stopping	Patience = 10 (based on validation balanced accuracy)
Evaluation Metrics	Accuracy, F1-score, AUC, Confusion Matrix
Model Checkpoints	Best balanced accuracy checkpoint

###### 2.2.4.1.1 (1) Classification head redesign

The original fully connected (FC) layer of ResNet-18, which outputs logits for 1,000 ImageNet classes, is replaced with a custom head tailored for binary classification. Specifically, this head consists of:

- A dropout layer with probability p=0.5 to introduce regularization and prevent overfitting on limited medical image data.
- A fully connected layer that projects the 512-dimensional feature vector to 2 output logits corresponding to the two classes.

###### 2.2.4.1.2 (2) Loss function with class weights

To address class imbalance, this study employs a CrossEntropyLoss function with:

- **Class-balancing weights**, computed using sklearn’s compute_class_weight based on the label distribution in the training set. These weights are passed to the loss function to mitigate bias towards the majority class, as formalised in Equation 1.

###### 2.2.4.1.3 Mathematical formulation

The weighted binary cross-entropy loss is defined as:

$$L_{\text{weighted}}=-\left[w_{1}\cdot y\cdot \log\;(\hat{y})+w_{0}\cdot (1-y)\cdot \log\;(1-\hat{y})\right]$$ (1)

where y∈{0,1} is the ground truth label, $\hat{y}\in [0,1]$ is the predicted probability, and $w_{0},w_{1}$ are class-specific weights.

###### 2.2.4.1.4 (3) Data augmentation strategy

To enhance generalization and reduce overfitting, this study applies a set of data augmentations during training:

- Resize to 224×224 to match the expected input size of ResNet-18.
- RandomHorizontalFlip to simulate left-right body variation.
- RandomRotation up to $\pm 10^{\circ }$ to account for positioning variability.
- ColorJitter (brightness, contrast = 0.2) to introduce photometric noise.
- Normalization using ImageNet mean and standard deviation.

For validation, only resizing and normalization are applied to ensure consistency in evaluation.

###### 2.2.4.1.5 (4) Optimization and training schedule

This study uses the AdamW optimizer with the following settings:

- Initial learning rate of $1\times 10^{-4}$.
- Weight decay regularization of $1\times 10^{-5}$.

The model is trained for up to 30 epochs with early stopping based on validation balanced accuracy, using a patience of 10 epochs to prevent overfitting.

###### 2.2.4.1.6 (5) Evaluation and checkpointing

Model performance is evaluated each epoch on a held-out validation set using:

- **Accuracy**, **F1-score**, and **ROC-AUC**.
- **Confusion matrix** analysis for error inspection.

The model achieving the highest validation balanced accuracy is saved as the best checkpoint.

###### 2.2.4.1.7 (6) Early stopping and model selection

Early stopping is triggered when validation balanced accuracy fails to improve for 10 consecutive epochs. The best-performing model is used for final evaluation on the held-out test set.

##### 2.2.4.2 Modified DenseNet-121

This study adopts DenseNet-121 as the backbone architecture for chest X-ray image classification. DenseNet introduces dense connectivity, where each layer receives inputs from all preceding layers. This design encourages feature reuse, strengthens gradient propagation, and has demonstrated excellent performance in various medical imaging tasks.

To adapt DenseNet-121 for our binary classification task (“diseased” vs. “no disease”), several customizations are applied:

- **Classifier modification:** The original classifier layer is replaced with a lightweight head consisting of a Dropout layer (p=0.5) followed by a fully connected layer mapping to two output logits. This design helps prevent overfitting and enables probabilistic interpretation via softmax activation.
- **Class-balanced loss:** This study incorporates a weighted CrossEntropyLoss, where class weights are computed based on the label distribution in the training set using sklearn’s compute_class_weight. This adjustment mitigates the effects of dataset imbalance between “diseased” and “no disease” samples.
- **Data augmentation:** To improve generalization, this study applies standard image augmentations during training, including resizing to 224×224, random horizontal flip, rotation (up to $\pm 10^{\circ }$), and color jittering (brightness and contrast). All images are normalized using ImageNet statistics to match the pretraining domain.
- **Learning strategy:** The model is trained using Adam optimizer with a learning rate of $5\times 10^{-5}$ and weight decay of $1\times 10^{-5}$. A step-based learning rate scheduler decays the learning rate by a factor of 0.5 every 5 epochs. Early stopping is employed with a patience of 10 epochs based on validation balanced accuracy.
- **Threshold optimization:** During validation, this study computes the optimal classification threshold using the Youden index, which maximises the difference between sensitivity and false positive rate Equation 2:
  $$t^{*}=\arg\;\max_{t}\left(\text{TPR}(t)-\text{FPR}(t)\right)$$ (2)
  where TPR(t) and FPR(t) denote the true positive rate and false positive rate at threshold t, respectively. The optimal threshold was constrained to the interval [0.4,0.7] to prevent degenerate threshold selection while maintaining clinically meaningful sensitivity and specificity. This adaptive threshold is used to convert model probabilities into binary predictions.
- **Evaluation and checkpointing:** At each epoch, this study monitored validation accuracy, F1-score, AUC, and balanced accuracy. The model achieving the highest balanced accuracy is saved as the best checkpoint. Final evaluation includes confusion matrix visualization and performance reporting.

This customized DenseNet-121 pipeline combines architectural strength with practical training strategies, yielding a robust classifier for binary chest X-ray diagnosis.

##### 2.2.4.3 Modified ConvNeXt-Tiny

ConvNeXt is a modern convolutional neural network architecture inspired by Vision Transformers (ViTs), designed to bridge the gap between traditional CNNs and transformer-based models. ConvNeXt-Tiny, in particular, is a lightweight variant that incorporates several architectural innovations: large kernel depthwise convolutions, GELU activations, layer normalization (instead of batch normalization), and inverted bottleneck structures. These components enable the model to learn hierarchical visual representations with fewer inductive biases, while maintaining strong performance on large-scale benchmarks such as ImageNet, despite its reduced parameter count.

In our implementation, ConvNeXt-Tiny is initialized with pretrained weights (ConvNeXt˙Tiny˙Weights.IMAGENET1K˙V1) and adapted for binary classification of chest X-ray images via the following modifications:

- **Classifier modification:** The final classification head is replaced by a linear layer mapping the pretrained feature dimension to two output logits. This layer directly replaces the original model.classifier[2] component of the pretrained model.
- **Class-balanced training:** To address class imbalance in the dataset, this study computes class weights from the training labels and incorporate them in a weighted CrossEntropyLoss function. This encourages the model to treat both classes more equally during gradient updates.
- **Data preprocessing:** This study adopts the default image transforms associated with the pretrained ConvNeXt-Tiny weights, which include resizing, center cropping, normalization, and other augmentations tailored to the model’s pretraining distribution.
- **Optimization and regularization:** The model is optimized using the AdamW optimizer with a learning rate of $1\times 10^{-4}$ and weight decay of $1\times 10^{-5}$. A validation-based early stopping mechanism with a patience of 10 epochs is employed to prevent overfitting.
- **Threshold calibration:** During validation, we use the ROC curve to compute the optimal decision threshold via Youden’s J statistic. The threshold is constrained to the interval [0.4,0.7] to prevent degenerate selection while preserving clinically meaningful sensitivity and specificity. This calibrated threshold is used to binarize softmax probabilities into final class predictions.
- **Evaluation and checkpointing:** At each epoch, we monitor standard classification metrics including accuracy, F1-score, AUC, and balanced accuracy. The model checkpoint achieving the highest balanced accuracy on the validation set is saved for final evaluation. Performance curves and confusion matrices are also generated for in-depth analysis.

This setup combines the architectural strengths of ConvNeXt-Tiny with practical training strategies tailored to the medical imaging domain, offering a strong balance between model capacity, efficiency, and classification robustness.

##### 2.2.4.4 Modified EfficientNet-B1

EfficientNet is a family of convolutional neural networks designed using compound scaling principles, which uniformly scales network depth, width, and input resolution in a balanced and principled way. This approach leads to better accuracy and efficiency trade-offs compared to traditional scaling strategies. The B1 variant increases input resolution and model capacity relative to the base model (B0), providing improved accuracy while remaining computationally tractable, making it particularly suitable for deployment in clinical workflows.

In our implementation, we adopt EfficientNet-B1 with ImageNet-pretrained weights and apply several customizations for binary classification of chest X-ray images:

- **Classifier adaptation:** The final fully connected layer (model.classifier[1]) is replaced with a linear layer outputting logits for the two target classes (“no disease” and “diseased”).
- **Loss function:** This study employs a class-weighted CrossEntropyLoss Class weights are computed from the training data distribution using sklearn’s compute_class_weight, addressing the inherent class imbalance.
- **Image preprocessing:** Images are resized to 240×240 to match the expected input resolution for EfficientNet-B1. During training, this study applied standard data augmentations including random horizontal flip, rotation ($\pm 10^{\circ }$), and color jittering. All images are normalized using ImageNet statistics.
- **Optimization and scheduling:** The model is trained using the AdamW optimizer with a learning rate of $1\times 10^{-4}$ and weight decay of $1\times 10^{-5}$.
- **Early stopping and checkpointing:** Validation balanced accuracy is monitored at each epoch, and training is stopped early if no improvement is observed for 10 consecutive epochs. The best-performing model (based on validation balanced accuracy) is saved for final evaluation on the held-out test set.
- **Evaluation metrics:** This study reports validation accuracy, F1 score, ROC-AUC, and plot confusion matrices to assess model performance in detail. Performance curves over epochs are also visualized to monitor training dynamics.

This configuration leverages the efficiency and scalability of EfficientNet-B1 while tailoring it to the specific demands of medical image classification tasks.

All models were initialised with ImageNet-pretrained weights and fine-tuned end-to-end on the chest X-ray dataset. The final classification layer in each model was replaced with a fully connected layer producing two logits, optionally preceded by a dropout layer (e.g., 0.5 for ResNet-18 and DenseNet-121). No layers were frozen during training, allowing the entire network to adapt to the medical imaging domain.

To address class imbalance, class weights were calculated using scikit-learn’s compute_class_weight and incorporated into the cross-entropy loss function. Optimisation was performed using either Adam or AdamW, with learning rates of $1\times 10^{-4}$ (ConvNeXt-Tiny, ResNet-18, EfficientNet-B1) or $5\times 10^{-5}$ (DenseNet-121), and weight decay of $1\times 10^{-5}$. For DenseNet-121, a StepLR scheduler was employed (step size of 5 epochs, decay factor 0.5).

Training was conducted for up to 30 epochs, with early stopping triggered if validation balanced accuracy did not improve for 10 consecutive epochs. Batch sizes were set to 16 or 20 depending on model size and GPU memory constraints.

To determine the optimal classification threshold, receiver operating characteristic (ROC) curves were computed for each model on the validation set. The threshold corresponding to the maximum Youden Index (J=TPR−FPR) was selected, constrained to the interval [0.4,0.7] to prevent degenerate threshold selection. Final evaluation was performed on the held-out test set using accuracy, F1-score, area under the ROC curve (AUC), sensitivity, specificity, and balanced accuracy.

This comprehensive approach enabled a comparative analysis of diverse CNN architectures under a consistent training framework, guided by weak supervision from large language model-derived labels.

#### 2.2.5 Model architectures and training configuration

The four CNN architectures evaluated were **ResNet18, DenseNet121, EfficientNet-B1, and ConvNeXt-Tiny**. All models were initialised with ImageNet-pretrained weights and fine-tuned on the CXR data.

Each model was trained using the AdamW optimiser (ConvNeXt-Tiny, ResNet-18, EfficientNet-B1; learning rate $1\times 10^{-4}$) or Adam optimiser (DenseNet-121; learning rate $5\times 10^{-5}$), with weight decay $1\times 10^{-5}$. Class-weighted binary cross-entropy loss was employed to counteract class imbalance. Training was performed for up to 30 epochs with early stopping based on validation balanced accuracy (patience of 10 epochs). For DenseNet-121, a StepLR scheduler was used (step size of 5 epochs, decay factor 0.5).

#### 2.2.6 Evaluation metrics

To comprehensively assess the performance of our binary classification models (*diseased* vs. *no disease*), this study reported a series of standard evaluation metrics commonly used in medical image analysis. These metrics capture not only overall correctness but also the model’s behavior on imbalanced data and its ability to distinguish between positive and negative cases.

- **Accuracy:** The proportion of correctly predicted samples among all predictions as given in Equation 3.
  $$\text{Accuracy}=\frac{\mathrm{TP}+\mathrm{TN}}{\mathrm{TP}+\mathrm{TN}+\mathrm{FP}+\mathrm{FN}}$$ (3)
- **Area Under the ROC Curve (AUC):** Measures the model’s ability to distinguish between the two classes across all classification thresholds. A higher AUC indicates better separability. This metric is threshold-independent and robust to class imbalance.
- **F1-Score:** The harmonic mean of precision and recall, representing a balance between false positives and false negatives as given in Equation 4. Particularly valuable in medical settings where both types of errors carry clinical risk.
  $$\text{F1}=2\cdot \frac{\text{Precision}\cdot \text{Recall}}{\text{Precision}+\text{Recall}}$$ (4)
- **True Positive Rate (TPR)/Sensitivity/Recall:** The proportion of actual positive cases that are correctly identified, as given in Equation 5. In clinical applications, high sensitivity is crucial to avoid missing diseased cases.
  $$\text{TPR}=\frac{\mathrm{TP}}{\mathrm{TP}+\mathrm{FN}}$$ (5)
- **True Negative Rate (TNR)/Specificity:** The proportion of actual negative cases correctly classified, as given in Equation 6. Specificity is important to prevent over-diagnosis and unnecessary intervention.
  $$\text{TNR}=\frac{\mathrm{TN}}{\mathrm{TN}+\mathrm{FP}}$$ (6)

All metrics are computed on the held-out test set, and threshold-based metrics (e.g., accuracy, F1, TPR, TNR) are evaluated using the calibrated threshold derived from the validation-set ROC curve via Youden’s Index. This ensures a balanced trade-off between sensitivity and specificity.

## 3 Results

This section presents the experimental design, training configuration, and comparative performance of four convolutional neural networks (CNNs) trained on chest X-ray images using weak supervision derived from large language model (LLM)-generated labels.

### 3.1 Experimental setup

**Figure 8:**
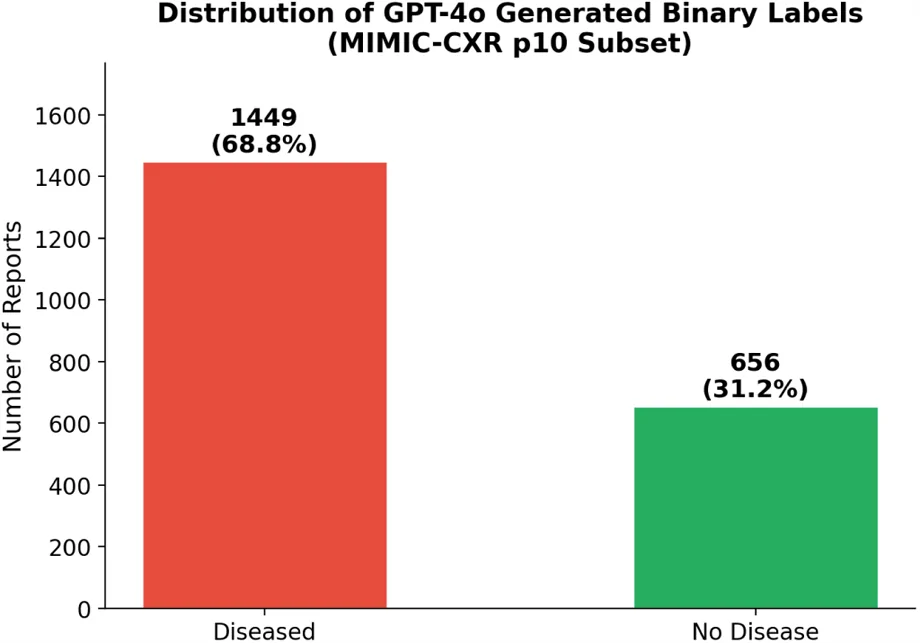
Distribution of GPT-generated binary labels.

The dataset consists of 2,097 chest radiograph studies with valid images from the MIMIC-CXR dataset p10 partition. Each study was assigned a binary label (diseased or no disease) based on a GPT-4o prompt applied to the associated free-text radiology report. Figure 8 shows the distribution of these binary labels. Approximately 68.8% of studies were labelled as diseased, and 31.2% as no disease, indicating moderate class imbalance.

### 3.2 LLM generated label quality

#### 3.2.1 LLM label evaluation

To evaluate the quality of LLM-generated labels, we compared them against the expert annotations described in the methodology. This evaluation provides insight into the effectiveness of using LLMs for weak supervision in medical imaging tasks.

- **Total Samples Evaluated:** 210 chest X-ray reports.
- **Ground Truth Source:** Annotated by a board-certified radiologist.
- **Ground Truth Distribution:** 126 diseased, 84 no disease.

The confusion matrix in Table 3 illustrates the agreement between LLM predictions and radiologist annotations.

**Table 3:** Confusion matrix: LLM vs. radiologist-annotated labels.

Actual/Predicted	Predicted Diseased	Predicted No Disease
Actual Diseased	114 (TP)	12 (FN)
Actual No Disease	3 (FP)	81 (TN)

Classification metrics for each class are summarised in Table 4.

**Table 4:** LLM label evaluation against radiologist ground truth.

Class	Precision	Recall	F1 Score	Support
Diseased	97.4%	90.5%	93.8%	126
No Disease	87.1%	96.4%	91.5%	84
Overall Accuracy	**92.9%**			

Bold value indicates the overall accuracy across all evaluated reports.

##### 3.2.1.1 Discussion

The LLM demonstrates high precision (97.4%) and recall (90.5%) in identifying diseased reports, indicating that it both rarely misclassifies healthy cases as diseased and catches the vast majority of true disease cases. For “no disease” reports, the model exhibits similarly strong performance with high recall (96.4%) and precision (87.1%). The expanded 210-report validation set, with a higher proportion of diseased cases (126 vs. 84), provides a robust estimate of label quality and confirms the effectiveness of the structured clinical prompt designed in collaboration with a radiologist.

Overall, the LLM achieves an excellent accuracy of 92.9%, and its balanced performance across both classes makes it a highly reliable tool for generating weak labels in downstream tasks. Future improvements may include ensemble LLMs or uncertainty-aware filtering to further enhance clinical utility.

### 3.3 Quantitative results

To assess model stability and provide confidence intervals, each CNN architecture was trained across five random seeds (42–46) with patient-level re-splitting, and the model was trained from random initialisation. Final evaluation was performed on the held-out test set using the optimal threshold determined on the validation set. Table 5 reports the mean and 95% confidence interval (CI) for each metric across the five seeds, using the t-distribution with 4 degrees of freedom. Table 6 provides the per-seed breakdown for all metrics.

**Table 5:** Test-set performance of CNN models across five random seeds (mean [95% CI]).

Model	AUC	Acc.	F1	Sens.	Spec.	Bal. Acc.
ResNet-18	0.822 [0.791, 0.853]	0.782 [0.746, 0.818]	0.840 [0.797, 0.884]	0.854 [0.758, 0.950]	0.616 [0.487, 0.744]	0.735 [0.712, 0.758]
DenseNet-121	0.808 [0.787, 0.828]	0.775 [0.728, 0.822]	0.837 [0.795, 0.879]	0.853 [0.797, 0.908]	0.608 [0.546, 0.671]	0.731 [0.694, 0.767]
EfficientNet-B1	0.797 [0.758, 0.835]	0.760 [0.719, 0.801]	0.821 [0.781, 0.862]	0.814 [0.756, 0.871]	0.641 [0.579, 0.703]	0.727 [0.701, 0.754]
ConvNeXt-Tiny	**0.832** [0.801, 0.863]	0.750 [0.686, 0.814]	0.808 [0.744, 0.872]	0.789 [0.628, 0.951]	**0.688** [0.511, 0.865]	**0.739** [0.708, 0.769]

Bold value indicates the best performance for each metric among the four models.

**Table 6:** Per-seed test-set performance of all CNN architectures across five random seeds.

Model	Seed	AUC	Acc.	F1	Sens.	Spec.	Bal. Acc.
ConvNeXt-Tiny	42	0.835	0.819	0.870	0.901	0.648	0.775
	43	0.856	0.685	0.739	0.607	0.901	0.754
	44	0.855	0.766	0.830	0.893	0.541	0.717
	45	0.811	0.763	0.827	0.844	0.597	0.720
	46	0.801	0.717	0.775	0.701	0.755	0.728
ResNet-18	42	0.807	0.796	0.851	0.867	0.648	0.758
	43	0.848	0.814	0.879	0.915	0.534	0.725
	44	0.844	0.764	0.817	0.823	0.659	0.741
	45	0.790	0.741	0.793	0.738	0.748	0.743
	46	0.820	0.794	0.862	0.927	0.490	0.709
DenseNet-121	42	0.809	0.755	0.814	0.796	0.669	0.732
	43	0.830	0.832	0.887	0.896	0.656	0.776
	44	0.807	0.757	0.818	0.857	0.582	0.720
	45	0.783	0.738	0.808	0.820	0.571	0.696
	46	0.808	0.794	0.858	0.895	0.563	0.729
EfficientNet-B1	42	0.808	0.755	0.810	0.776	0.711	0.743
	43	0.834	0.818	0.878	0.893	0.611	0.752
	44	0.805	0.740	0.796	0.793	0.647	0.720
	45	0.751	0.738	0.807	0.816	0.580	0.698
	46	0.785	0.749	0.814	0.791	0.656	0.723

**ConvNeXt-Tiny** achieved the highest mean AUC (0.832, 95% CI [0.801, 0.863]) and the highest balanced accuracy (0.739). Paired t-tests on AUC across seeds revealed that ConvNeXt-Tiny significantly outperformed EfficientNet-B1 (ΔAUC=0.035, t=4.15, p=0.014) and showed a borderline advantage over DenseNet-121 (ΔAUC=0.024, t=2.74, p=0.052). The difference between ConvNeXt-Tiny and ResNet-18 was not statistically significant (ΔAUC=0.010, t=1.20, p=0.297).

**ResNet-18** demonstrated competitive AUC (0.822) and the highest mean F1-score (0.840), but exhibited higher variability in specificity across seeds (SD=0.103), indicating less stable performance on negative cases.

**DenseNet-121** showed the most consistent sensitivity across seeds (SD=0.045), making it a reliable choice when high recall is prioritised.

**EfficientNet-B1** yielded the lowest AUC (0.797) among the four architectures, although its specificity (0.641) was competitive with ResNet-18 and DenseNet-121.

All models achieved mean AUC values above 0.79, confirming that the GPT-4o-derived weak supervision signal was sufficient for effective training across diverse architectures. The confidence intervals for sensitivity and specificity were notably wider than those for AUC, reflecting the inherent trade-off modulated by the decision threshold A condensed summary of the four models' performance is presented in Table 7.

**Table 7:** Summary of CNN performance on test set (mean across 5 seeds [95% CI]).

Model	F1	AUC	Sens.	Spec.
ResNet-18	0.840 [0.797, 0.884]	0.822 [0.791, 0.853]	0.854 [0.758, 0.950]	0.616 [0.487, 0.744]
DenseNet-121	0.837 [0.795, 0.879]	0.808 [0.787, 0.828]	0.853 [0.797, 0.908]	0.608 [0.546, 0.671]
EfficientNet-B1	0.821 [0.781, 0.862]	0.797 [0.758, 0.835]	0.814 [0.756, 0.871]	0.641 [0.579, 0.703]
ConvNeXt-Tiny	0.808 [0.744, 0.872]	**0.832** [0.801, 0.863]	0.789 [0.628, 0.951]	**0.688** [0.511, 0.865]

Bold value indicates the best performance for each metric among the four models.

### 3.4 Confusion matrices and error analysis

**Figure 9:**
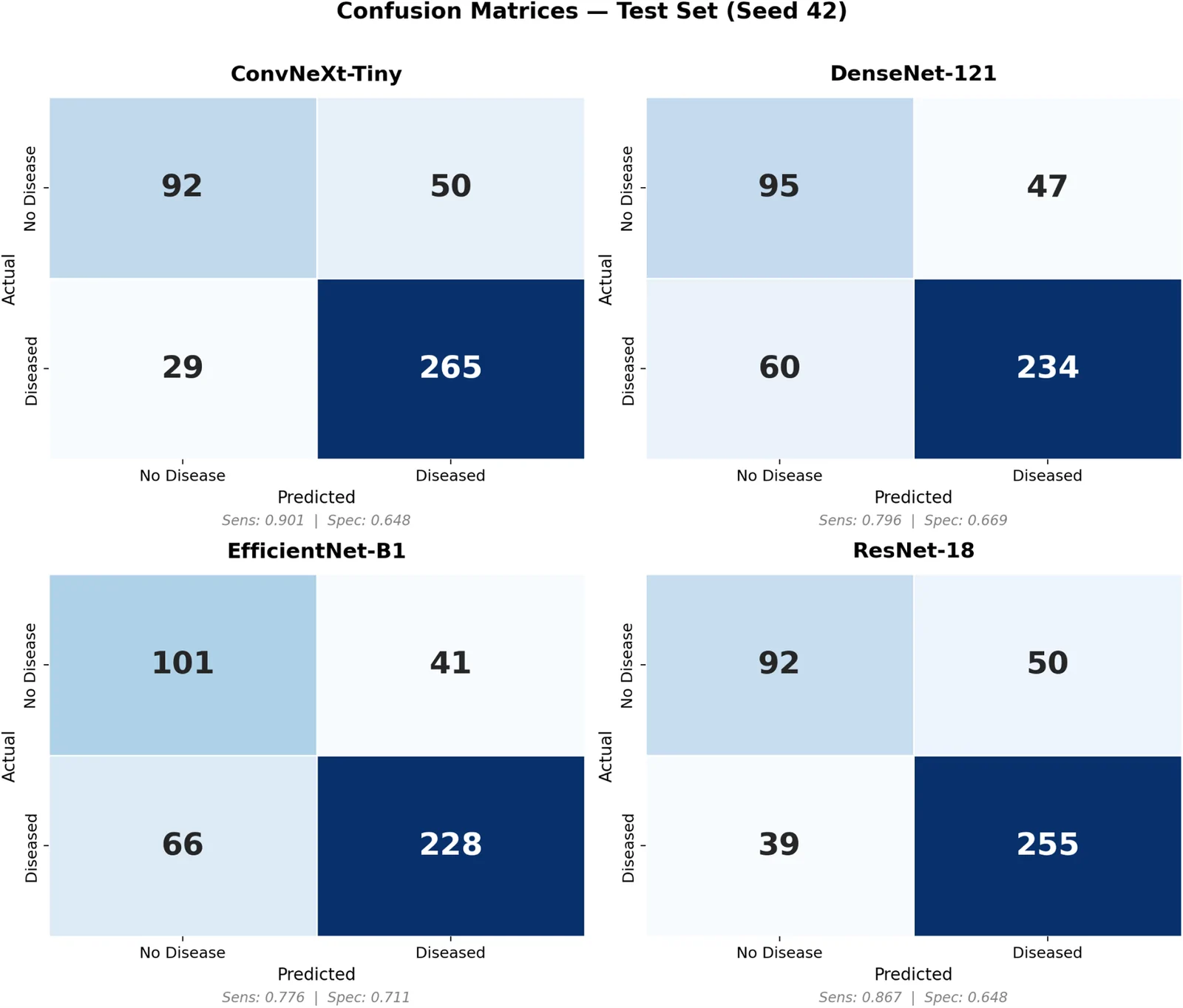
Confusion matrices for the four CNN models: ResNet18, DenseNet121, EfficientNet-B1, and ConvNeXt-Tiny.

Confusion matrices were generated for all four CNN architectures to examine classification behaviour in detail, as presented in Figure 9 for a representative seed. Across the five seeds, several consistent patterns emerged.

**ConvNeXt-Tiny** exhibited the lowest mean false positive count, consistent with its higher specificity (0.688). However, its sensitivity (0.789) was the lowest among the four models, with correspondingly higher false negatives. This indicates a conservative decision boundary that prioritises specificity at the expense of recall—a trade-off that may be appropriate in screening scenarios where false alarms are costly, but requires careful consideration in settings where missed pathology carries high clinical risk.

**DenseNet-121** and **ResNet-18** both achieved higher sensitivity (0.853–0.854), translating to fewer false negatives, but at the cost of lower specificity (0.608–0.616). This behaviour reflects a more permissive threshold that flags more studies as diseased. Such a configuration may be preferable in triage applications where the priority is to avoid missing any positive case.

**EfficientNet-B1** occupied an intermediate position, with moderate sensitivity (0.814) and specificity (0.641), resulting in the most balanced error profile among the four architectures, albeit with the lowest overall AUC.

The variability in confusion matrix patterns across seeds, particularly for ConvNeXt-Tiny (sensitivity SD=0.130, specificity SD=0.143), underscores the sensitivity of threshold-dependent metrics to the data split. This reinforces the importance of the multi-seed evaluation framework and the reporting of confidence intervals, as threshold-independent metrics such as AUC showed considerably lower variability.

## 4 Discussion

### 4.1 Overview

This study explored the use of large language models (LLMs), particularly GPT-4o, to generate binary disease labels from chest X-ray radiology reports. These automatically generated labels were then used to train convolutional neural networks (CNNs) for image classification. The overarching aim was to assess whether supervision from generative AI could approximate manually curated annotations for medical image analysis. The findings demonstrate that this approach can yield competitive performance, suggesting the feasibility of integrating natural language processing with computer vision in a unified diagnostic framework.

### 4.2 Review of methodology

The study employed GPT-4o to extract disease presence information from unstructured radiology reports, thereby generating binary labels without the need for manual annotation. These labels were then used to train four convolutional neural network (CNN) architectures: ResNet18, DenseNet121, EfficientNet-B1, and ConvNeXt-Tiny. A patient-level split (70/10/20) was used to prevent data leakage, and the test-set performance of these models was assessed across five random seeds using standard classification metrics, including accuracy, F1-score, AUC, sensitivity, specificity, and balanced accuracy, with 95% confidence intervals computed via the t-distribution.

To evaluate the quality of the LLM-generated labels, a set of 210 chest X-ray reports was manually annotated by a board-certified radiologist to serve as ground truth. This set comprised 126 diseased cases and 84 with no disease. The GPT-generated labels were then compared against these expert annotations using standard metrics, including precision, recall, F1-score, and overall accuracy.

The evaluation showed that the LLM achieved high precision (97.4%) and recall (90.5%) in identifying diseased cases, indicating strong performance in both avoiding false alarms and capturing true disease. For the “no disease” class, recall was high (96.4%) with good precision (87.1%), suggesting a well-balanced classifier. The overall accuracy reached 92.9%, with a confusion matrix revealing 114 true positives, 12 false negatives, 3 false positives, and 81 true negatives. These results indicate a high level of agreement with expert annotation and strongly support the feasibility of using LLM-generated labels as weak supervision signals for downstream model training.

### 4.3 Discussion of quantitative results

The quantitative evaluation across four CNN architectures trained with LLM-generated labels reveals several significant insights.

#### 4.3.1 LLM label quality

The GPT-4o model achieved an overall accuracy of 92.9% when its labels were compared against expert annotations on the full 210-report validation set. The precision for the diseased class reached 97.4%, indicating that nearly all positive predictions were correct, while the recall of 90.5% shows that most diseased cases were captured. For the no disease class, the model achieved high recall (96.4%) and good precision (87.1%), reflecting balanced performance with a low false positive rate. These values are reflected in the class-specific F1-scores: 93.8% for diseased and 91.5% for no disease. After further investigation into the quality of the difference between the LLM-generated labels and the radiologist’s labels, it became clear that the human expert disregarded non-significant clinical abnormalities.

#### 4.3.2 Model performance

All four CNN models trained on LLM-generated labels achieved mean AUC scores exceeding 0.79 on the held-out test set, confirming that the weak supervision signal was sufficient for effective training. ConvNeXt-Tiny achieved the highest mean AUC (0.832) and specificity (0.688), significantly outperforming EfficientNet-B1 in AUC (paired t-test, p=0.014) and showing a borderline advantage over DenseNet-121 (p=0.052). Its lower sensitivity (0.789) reflects a conservative decision threshold that prioritises specificity—a configuration that may be preferable in screening scenarios where false positives carry high downstream costs. ResNet-18 demonstrated the highest mean F1-score (0.840), while DenseNet-121 showed the most stable sensitivity across seeds (SD=0.045). EfficientNet-B1, despite the lowest AUC, maintained competitive specificity (0.641) with lower variability.

#### 4.3.3 Sensitivity vs. specificity trade-off

The results reveal a clear architectural spectrum in the sensitivity–specificity trade-off. DenseNet-121 and ResNet-18 are sensitivity-dominant, making them suitable for triage applications where minimising false negatives is paramount. ConvNeXt-Tiny is specificity-dominant, favouring applications with high costs associated with false positives. EfficientNet-B1 occupies an intermediate position with a more balanced trade-off. The width of the confidence intervals for sensitivity and specificity, particularly for ConvNeXt-Tiny (sensitivity SD=0.130, specificity SD=0.143), highlights the importance of the multi-seed evaluation: a single run could misleadingly suggest either excellent or poor threshold-dependent performance.

#### 4.3.4 Multi-seed stability and confidence intervals

The use of five random seeds revealed that AUC—a threshold-independent metric—was the most stable across runs (mean coefficient of variation = 3.2%), while sensitivity and specificity showed substantially higher variability (CV = 5.7%–20.7%). This finding underscores the importance of reporting confidence intervals for threshold-dependent metrics in medical imaging studies, and supports the use of AUC as the primary metric for model comparison when the clinical decision threshold has not been externally validated.

These quantitative findings validate the feasibility of using LLM-generated supervision for training CNNs in medical imaging and highlight the importance of architecture selection based on task-specific requirements.

### 4.4 Results and comparison with existing literature

Among the evaluated models, ConvNeXt-Tiny achieved the highest AUC and balanced accuracy on the held-out test set, while ResNet-18 showed competitive AUC and the highest F1-score. These results are consistent with prior studies that demonstrated the effectiveness of CNNs in chest X-ray classification (9). Moreover, our work extends previous efforts by integrating LLM-based label generation, as suggested in recent literature (28), but goes further by completing a full pipeline from clinical text to image-level model training with rigorous multi-seed evaluation. Unlike prior work that focused primarily on language tasks or required human-in-the-loop verification, this study demonstrates a fully automated supervision strategy with quantified uncertainty.

### 4.5 Summary of contributions

- Demonstrating the feasibility of using GPT-4o with a structured clinical protocol to generate weak supervision labels from clinical text.
- Evaluating the performance of four CNN architectures under LLM-derived supervision using a patient-level split and multi-seed evaluation with 95% confidence intervals.
- Quantifying model stability and statistical significance through paired t-tests, revealing ConvNeXt-Tiny’s significant advantage over EfficientNet-B1 (p=0.014).
- Assessing the trade-offs between scalability and reliability in automated clinical pipelines.

### 4.6 Limitations

Despite promising outcomes, several limitations must be acknowledged. Firstly, GPT-4o does not have access to image content or structured patient metadata (in the configuration used here), which may introduce label noise. Secondly, the study was conducted using data from a single institution, potentially limiting generalisability. Thirdly, the binary classification setup oversimplifies the diagnostic landscape, where multiple co-existing conditions and severity levels are often present. Fourthly, while the multi-seed evaluation provides confidence intervals, the sample size of five seeds limits the precision of variance estimates, and the wide CIs for sensitivity and specificity suggest that threshold-dependent metrics should be interpreted with caution.

### 4.7 Future works

Future work could focus on extending the pipeline to multi-label classification, incorporating uncertainty estimation in label generation, and exploring multimodal LLMs that can directly process both images and text. The completed 210-report gold-standard validation set has strengthened the statistical rigour of the LLM label quality evaluation; further increases in seed count and validation set size, as well as validation across diverse institutions and clinical settings, remain important directions for enhancing robustness and scalability.

### 4.8 Conclusion

The novelty of our approach lies in the fact that label creation requires substantial clinical time to construct robust data preprocessing for a deep learning system from free-text radiology reports. Our approach is to use a clinically informed and verified prompt on LLMs to generate labels. Label validation was conducted on a 10% subset of the entire dataset. The statistical soundness of the CNN was verified using performance scores, including confidence intervals. The clinical relevance lies in the usage of the model as a screening/triage system. This is particularly useful in a low-resource setting where radiologists are not readily available, but a clinical urgency needs to be indicated. The usefulness is demonstrated by the inclusion of clinical relevance in the definitions of the labels. When applied as a clinical decision support system (CDSS), it could reduce radiologists’ workload and enable early detection of clinically relevant findings in chest X-ray images, while keeping the human in the loop (the radiologist) as the final provider of the diagnosis. The approach’s explainability lies in the fact that the prompt for label generation is fully documented in the methodology.

## Data Availability

Publicly available datasets were analyzed in this study. This data can be found here: https://physionet.org/content/mimic-cxr/2.1.0/.
